# Development of the Method for Determination of Volatile Sulfur Compounds (VSCs) in Fruit Brandy with the Use of HS–SPME/GC–MS

**DOI:** 10.3390/molecules25051232

**Published:** 2020-03-09

**Authors:** Urszula Dziekońska-Kubczak, Katarzyna Pielech-Przybylska, Piotr Patelski, Maria Balcerek

**Affiliations:** Institute of Fermentation Technology and Microbiology, Faculty of Biotechnology and Food Sciences, Lodz University of Technology, Wolczanska 171/173, 90-924 Lodz, Poland; katarzyna.pielech-przybylska@p.lodz.pl (K.P.-P.); piotr.patelski@p.lodz.pl (P.P.); maria.balcerek@p.lodz.pl (M.B.)

**Keywords:** volatile sulfur compounds, VSCs, fruit brandy, HS–SPME, GC–MS

## Abstract

Volatile sulfur compounds (VSCs) play an important role in the aroma profile of fermented beverages. However, because of their low concentration in samples, their analysis is difficult. The headspace solid-phase microextraction (HS–SPME) technique coupled with gas chromatography and mass spectrometry (GC–MS) is one of the methods successfully used to identify VSCs in wine and beer samples. However, this method encounters more obstacles when spirit beverages are analyzed, as the ethanol content of the matrix decreases the method sensitivity. In this work, different conditions applied during HS–SPME/GC–MS analysis, namely: ethanol concentration, salt addition, time and temperature of extraction, as well as fiber coating, were evaluated in regard to 19 sulfur compounds. The best results were obtained when 50/30 μm Divinylbenzene/Carboxen/Polydimethylsiloxane (DVB/CAR/PDMS) was used to preconcentrate the analytes from the sample at 35 °C for 30 min. The dilution of samples to 2.5% *v*/*v* ethanol and the addition of 20% *w*/*v* NaCl along with 1% EDTA significantly improves the sensitivity of extraction. The optimized method was applied to three fruit brandy samples (plum, pear, and apple) and quantification of VSCs was performed. A total of 10 compounds were identified in brandy samples and their concentration varied greatly depending on the raw material used from production. The highest concentration of identified VSCs was found in apple brandy (82 µg/L).

## 1. Introduction

Volatile sulfur compounds (VSCs) are known to have a great impact on the aroma of various food and beverages, especially because of their abundance and low detection threshold. Their presence plays an important role in the aroma of foods such as vegetables, cheese, coffee, chocolate, UHT milk, as well as wine and beer [1]. In fermented beverages, sulfur compounds are formed as a result of both enzymatic (yeast fermentation) and nonenzymatic (chemical and thermal reactions during production process) pathways, and most of them are generally responsible for off-flavors in final products (rotten eggs, burnt rubber, cooked cabbage, etc.) [2,3]. Especially, VSCs are in the main area of interest of wine and beer producers as they often cause aroma defects in the final products [3,4,5]. The presence of VSCs in distilled beverages has not been studied in such a great manner as it has been for wine and beer.

Distilled (or spirit-based) beverages are produced worldwide from a wide variety of raw materials and include among others whiskey, rum, tequila, vodka, and brandy. Among them, brandies (both grape brandies and fruit brandies) are receiving increasing interest because of their unique flavor characteristic. Fruit brandies are produced by fermentation of fresh fruits or fruit mash and subsequent distillation of fermented mash at less than 86% *v/v* alcohol content to maintain the aroma and flavor of used raw material. Often, after distillation, the maturation (aging) of distillates in wooden casks is carried out. The maturation lasts from several months to several years, and the final product gains characteristic flavor dependent on the maturation conditions [6,7,8]. Depending on the used raw material, brandy’s name is preceded by the name of the fruit used in the production, e.g., apple brandy (e.g., Calvados), plum brandy (e.g., Slivovitz, Zwetschgenwasser), cherry brandy (e.g., Kirsch), or pear brandy [6,9].

The determination of VSCs in fermented beverages is difficult due to their low concentration in analyzed samples, and, for that reason, selecting the appropriate method of sample preconcentration is often necessary. The techniques that could be used to extract the volatile compounds from samples are liquid–liquid extraction (LLE) [10], static [11] or dynamic [12] headspace, stir bar sorptive extraction (SBSE) [13], vacuum distillation [14], solvent-assisted flavor evaporation (SAFE) [15], counter-current supercritical fluid extraction [16], and solid-phase microextraction (SPME) [4,17]. Among them, the SPME method has gained greater attention and has been successfully used in the determination of volatiles from solid [17], liquid [18], and gaseous samples [19]. SPME can be performed by either direct sampling (DI-SPME) or by analyzing the headspace above the sample (HS-SPME) [20].

SPME is an inexpensive, simple, and solvent-free technique of extraction of volatiles from the sample, and what is more, this method has good reproducibility and easily can be automated [20]. However, the appropriate experimental conditions must be first settled to ensure the effective extraction of the compounds of interest. The parameters that are crucial for SPME analysis are the selection of fiber (its polarity and thickness), extraction time and temperature, addition of soluble salts (sodium chloride, sodium hydrogencarbonate, potassium carbonate), agitation of the sample, and the concentration of analyte in the sample [20]. In case of analysis of samples containing alcohol (beer, wine, spirits) the concentration of ethanol has a great impact on the sensitivity of analysis when sulfur compounds are taken into consideration [21,22,23].

In this work, the influence of such parameters as the concentration of ethanol, addition of sodium chloride and EDTA to the sample prior to extraction, time and temperature of the process, as well as 5 different fiber coatings were analyzed with regard to 22 volatile sulfur compounds that can be found in fermented beverages. The selection of VSCs to be examined in this work was based on their content in alcoholic beverages including beer [4,21] and wine [4,24], as well as based on scarce data regarding spirit beverages, i.e., whisky [4], Calvados [10,25], and Cognac [10]. The selected conditions were applied to real fruit brandy samples to identify the present VSCs.

## 2. Results

### 2.1. Optimization of Solid-Phase Microextraction (SPME) Parameters

#### 2.1.1. Selection of Fiber Coating

**Figure 1:**
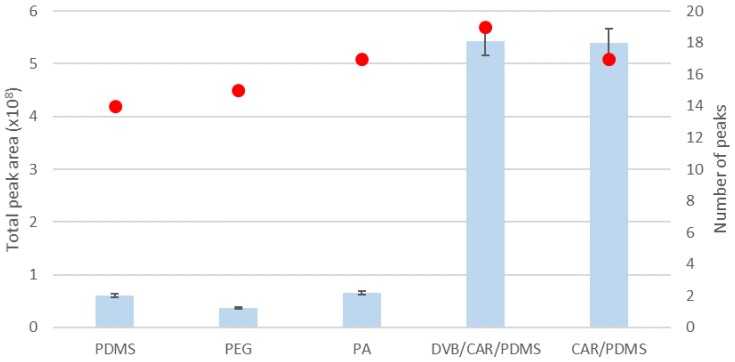
The results of SPME/GC–MS analysis of different fiber coatings (SPME extraction at 50 °C for 15 min). PDMS—100 μm Polydimethylsiloxane, PEG—60 µm Carbowax/Polyethylene Glycol, PA—85 μm Polyacrylate, CAR/PDMS—85 μm Carboxen/PDMS, Divinylbenzene/Carboxen/Polydimethylsiloxane (DVB/CAR/PDMS)—50/30 μm DVB/Carboxen/PDMS. Bars represents the peak area and dots represents number of detected peaks.

In the first stage of experiments, the selection of fiber coating was performed to obtain the best signals for tested VSCs. Five different coatings were evaluated: 100 μm PDMS, 60 µm PEG, 85 μm PA, 85 μm CAR-PDMS, and 50/30 μm DVB/CAR/PDMS. The most important parameters considered during selection of fiber are the thickness of the film and its polarity. Fibers covered with thicker film require a longer time to achieve extraction equilibrium but may provide higher sensitivity because of the greater mass of extracted analytes. Moreover, it is recognized that volatile compounds require a thick coating, and for semi-volatile compounds, a thin coating is more effective [20]. According to the literature, the mostly commonly used fibers for extraction of VSCs are CAR/PDMS [4,21,22] and DVB/CAR/PDMS [2,26,27], but other coatings were also tested. The ethanolic solution (5% *v/v*) of stock standard mixture in the total volume of 10 mL in 20 mL vials was preincubated at 50 °C for 15 min and then extraction took place at the same temperature for 15 min. After the GC–MS analysis, the total peak area as well as number of identified peaks were calculated, and the results are presented in Figure 1. The highest peak areas were obtained for both CAR/PDMS and DVB/CAR/PDMS fibers; however, we were not able to identify ethanethiol and dimethyl sulfide using the CAR/PDMS fiber, as these components were covered by ethanol peak. Therefore, the DVB/CAR/PDMS fiber was selected for further experiments. Interestingly, regardless of the fiber coating used for analysis, the presence of 2-(methylthio)ethanol, 3-(methylthio)propionaldehyde, and 3-(methylthio)-1-propanol was not established, although these compounds were present when direct injection of the standard mixture was performed (data not shown). This may be caused by the low affinity of these compounds for fiber coatings or their degradation under the conditions used; however, further work is needed.

#### 2.1.2. Effect of Ethanol and Sodium Chloride Concentration

In the next step of the experiments, the concentration of ethanol as well as addition of NaCl to the samples were evaluated. Three levels of ethanol content (% *v*/*v*) and four of NaCl dose (% *w*/*v*) in samples was analyzed, respectively, 2.5, 5, and 10, and 0, 10, 20, and 30. The results are displayed in Table 1. It is clear that with the increasing ethanol content in the samples, the area of peaks decreased, especially for thiols (1-propanethiol, 1-butanethiol, 1-pentanethiol). The less sensitive to the increasing ethanol content were dibutyl sulfide and dipropyl sulfide. The influence of the ethanol content in samples has been investigated to determine the extent to which the extraction is disturbed by higher alcoholic strength of the solution. This is particularly important when spirit beverages are to be analyzed, as the reduction of alcohol concentration is inextricably linked to the dilution of the remaining analytes present in the sample, including VSCs.

The ethanol content is one of the most important parameters to consider when sulfur compounds are extracted from fermented beverages. Recent studies conducted by Davis and Qian [22] showed that a sharp decrease in detectability of VSCs occurs when ethanol is present in the sample even at 0.5% *v*/*v* concentration. The authors also concluded that the alcoholic strength of the tested sample should be between 2%–4% *v*/*v* of ethanol to obtain reliable results. Additionally, Campillo et al. [4], when studying VSCs in beer, wine, and whisky, stated that the lowest detection limits can be obtained when the sample:water ratio is 5:15 and 2:18, respectively, for wine and whisky, which corresponds to the ethanol concentration of approximately 5% *v*/*v* (similar to that in beer).

The addition of sodium chloride to the samples is performed to increase the ionic strength of analytes and enhance the recovery rate due to the “salting-out” effect [20]. The literature gives conflicting information about whether this is a favorable stage or not for SPME, e.g., Campillo et al. [4] stated that the presence of sodium chloride in the amount of 0–1.5 g/5 mL of beer sample did not enhance the extraction of VSCs. On the other hand, many authors [22,24] report application of saturated salt during extraction as a factor increasing the ionic strength of analytes. In this study, four concentrations of NaCl, i.e., 0%, 10%, 20%, and 30% *w*/*v* were tested in terms of the VSCs adsorption during SPME. Most of the analyzed compounds achieved the highest peak areas at 20% *w*/*v* sodium chloride concentration (Table 1), and further increasing the salt concentration led to a decrease in the obtained signals. Similar results were obtained by Camara et al. [28], where HS–SPME was optimized to analyze terpenoids in Madera wines. Authors reported that the addition of salt up to 30% improves extraction of the majority of analytes, while a decrease was observed at 40% concentration. In this work, 2-methyltetrahydrothiophene-3-one, 2-thiophenecarboxyaldehyde, ethyl 3-(methylthio)propionate, and benzothiazole were the only analytes for which the highest response was obtained at 30% NaCl addition. Different results were obtained also for thiophenol, where the addition of salt significantly decreased the peak area, regardless of the salt concentration (approximately 80%–85% lower peak areas). What is more, the presence of ethanethiol and dimethyl sulfide was reported only in samples with 2.5% *v*/*v* of ethanol and with NaCl concentration up to 20% *w*/*v*. In every other sample, the peak of ethanol made identification of these components impossible.

**Figure 2:**
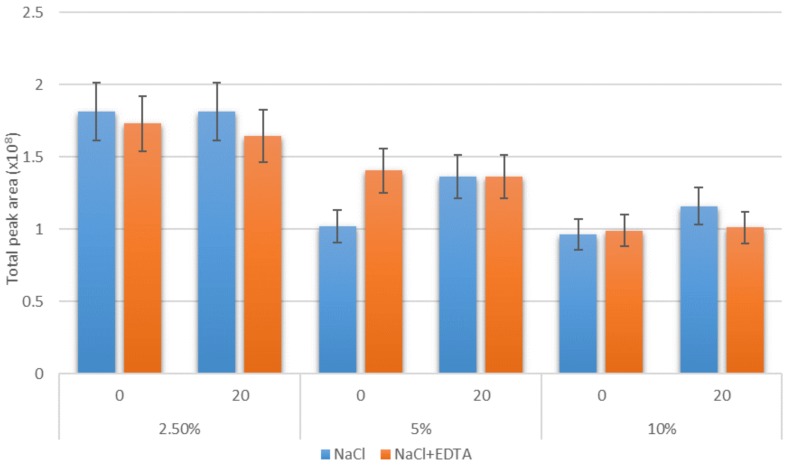
The effect of the addition of EDTA on volatile sulfur compounds (VSCs) extraction without (0% *w*/*v*) and with (20% *w*/*v*) NaCl treatment at different alcohol concentrations (2.5%, 5%, and 10% *v*/*v*). Extraction temperature: 50 °C, extraction time: 15 min; SPME fiber: DVB/CAR/PDMS.

One of the techniques used to improve SPME efficiency is the addition of EDTA to the samples [24]. According to Tikunov et al. [29], this procedure stabilizes the matrix by preventing metal-catalyzed oxidation of the compounds and increasing the pH. Considering the best results were obtained when 20% *w*/*v* NaCl addition was used, this concentration was chosen to access the effect of EDTA addition on the extraction efficiency. Moreover, the addition of EDTA to the samples without salt addition was investigated to check whether the exclusive addition of EDTA would increase the performance of SPME to the extent that no salt treatment would be necessary. It was previously stated that, especially in the case of the DI-SPME method, the addition of salt to the sample can shorten the lifetime of the fiber [20]. Figure 2 presents the effect of EDTA on the SPME absorption of VSCs tested in this study. The addition of EDTA did not change the total peak area of tested compounds and also resulted in the improvement in peak shape. Samples to which EDTA was added were characterized by sharper peaks, and what is more, the peak of thiophenol reach area was close to the one obtained when no addition of NaCl was used (approx. 1.5 × 10^8^).

Thus, in the next stages of experiments, the 2.5% *v*/*v* ethanol concentration and addition of 2 g NaCl along with 0.1 g EDTA to 10 mL of sample was selected.

#### 2.1.3. Effect of Extraction Time and Temperature

**Figure 3:**
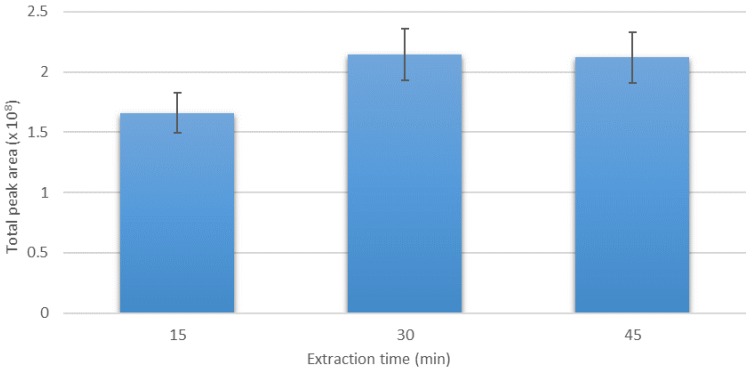
Effect of extraction time on the total peak area of analyzed VSCs. Ethanol concentration 2.5% *v/v*; NaCl addition 20% *w*/*v*; EDTA addition 1% *w*/*v*; extraction temperature: 50 °C; and SPME fiber: DVB/CAR/PDMS.

The time of extraction is one of the most important parameters during the SPME procedure. Three different extraction times were analyzed, i.e., 15, 30, and 45 min. The extraction was carried using DVB/CAR/PDMS fiber in 2.5% *v/v* ethanol solution, with the addition of 20% *w*/*v* of NaCl and EDTA in the total sample volume of 10 mL. The temperature of extraction was set at 50 °C. As can be seen from Figure 3, the total peak area increased significantly for 30 min extraction time when compared to 15 min. Further extension of the exposure time to 45 min did not significantly increase the sensitivity of extraction but only extended the overall analysis time.

In order to estimate the influence of the temperature of extraction on the efficiency of the SMPE process, five distinct temperatures were evaluated, namely: 20, 35, 50, 65, and 75 °C. The preconcentration was conducted with use of DVB/CAR/PDMS fiber in 2.5% *v*/*v* ethanol solution enriched in NaCl (20% *w*/*v*) and EDTA for 30 min, as was established in previous experiments. The obtained results are presented in Table 2. It can be seen that the best overall results were obtained when the temperature of 35 °C was used. The extraction at lower temperature (20 °C) gave higher signal response for lower boiling compounds (ethanethiol, dimethyl-sulfide, 1-propanethiol, thiophene, diethyl-sulfide, and 1-butanethiol) (Table 2), while the rest of compounds exhibited higher signals at elevated temperatures (mostly 35 or 50 °C). These results are in agreement with those reported by Campillo et al. [4], where the sensitivity of adsorption of dimethyl sulfide, diethyl sulfide, and dimethyl disulfide decreased with increasing the temperature in the range of 25–75 °C. In this study, application of temperatures above 50 °C (i.e., 65 and 75 °C) resulted in a stark decrease in the obtained peak areas, which may be due to formation of artefacts caused by thermal degradation.

Another important issue when VSCs are taken into consideration is the formation of artifacts during SMPE analysis coupled to GC–MS detection. The research conducted on *Allium* plants shows that some of the compounds detected by the SPME/GC–MS technique were not present in the original sample but were formed during analytical procedures as artifacts [30,31,32]. Sulfur compounds are often unstable and reactive and can undergo transformation to other compounds, e.g., methanethiol can be oxidized to dimethyl disulfide and dimethyl sulfide to dimethyl sulfoxide [33]. Several solutions were proposed to overcome this problem. Locatelli et al. [30] reported that the use of SPME coupled to liquid chromatography with UV detection allowed them to avoid thermal degradation of the compounds present in raw, cooked, and distilled garlic samples on the injector. Siebert et al. [34] applied the use of static headspace injection and cool-on-column gas chromatography coupled with sulfur chemiluminescence detector to analyze 68 various samples of Australian wines in which reductive aroma was observed. The authors were focused on such compounds as hydrogen sulfide, methanethiol, ethanethiol, methyl thioacetate, ethyl thioacetate, dimethyl sulfide, diethyl sulfide, carbon disulfide, dimethyl disulfide, and diethyl disulfide. Among them, only ethyl thioacetate and diethyl disulfide were not detected in any of 68 tested samples. The results obtained by Siebert et al. clearly indicate that those compounds are present in fermented beverages and are not only the artifacts formed during analytical procedures.

### 2.2. Method Validation

When the best SPME conditions were selected, the method was validated with respect to limits of detection and quantification, linearity range, and recovery. Namely, 10 mL of 2.5% ethanolic solution was placed in 20 mL vial along with 2 g NaCl and 0.1 g EDTA, then 10 µL of each standard working solution and internal standard (IS) (final concentration of IS was 0.09 µg/L) was added to each vial. Samples were extracted using DVB/CAR/PDMS fiber at 35 °C for 30 min. Analyses were performed in three replicates, and the obtained results are presented in Table 3. Calibration curves were prepared in the range from approximately 0.08 to 80 µg/L, depending on the analyte, by plotting the response from GC–MS against its concentration. Limit of quantification (LOQ) was established based on the standard deviation of the response and the slope of the calibration curve. Limit of detection (LOD) was calculated as one third of LOQ. Both LOQ and LOD were calculated with use of Agilent MassHunter Workstation Software Quantitative Analysis. For most of the studied compounds, the regression coefficient was above 0.99, with the exception of 1-propanethiol, 1-butanethiol, and 1-penthanethiol. For these compounds, the linearity was poorer (0.93–0.98). The calculated values of LOD and LOQ were in range of 0.001–0.171 µg/L and 0.002–0.569 µg/L, respectively. These concentrations are in most cases low enough to calculate the concentration of VSCs in real samples.

The recovery experiments were carried out by spiking the analytes to real samples (pear, plum, and apple brandy, each in triplicate) and further extraction under optimized conditions. The value of recovery percentage was calculated from the difference between the concentration of the analyte in the spiked and nonspiked samples divided by the added concentration of each compound [35]. The results (Table 3) shows satisfactory accuracy (recovery in range of 90–110%) for most compounds.

Higher error was obtained in the cases of 1-propanethiol (75–176%), 1-butanethiol (87–133%), and 1-pentanethiol (86–129%)—the same compounds that were characterized by poorer linearity. Additionally, the recovery percentage of dimethyl sulfide was unsatisfactory (78–107%), as this compound was found to undergo the chemical oxidation to dimethyl sulfoxide under adverse analysis conditions [1]. Moreover, for thiophenol and benzothiazole, the recovery rate was always higher than 100%.

### 2.3. Analysis of Real Samples

When the best parameters of the extraction were established, the analysis of commercial plum brandy, pear brandy, and apple brandy was performed to determine the VSCs content in samples. Before the extraction, the 10 mL of diluted samples (to 2.5% *v*/*v* ethanol content) were placed in a 20 mL vial along with 2 g of NaCl and 0.1 g EDTA and 10 µL of IS (thiophene). The SPME procedure was conducted with use of DVB/CAR/PDMS fiber at 35 °C for 30 min. Each sample was analyzed in triplicate in both SCAN and SIM mode. The chromatograms obtained in SCAN mode were used to identify the compounds by means of retention time and mass spectra and to perform the deconvolution of obtained GC–MS spectra and compare them with entries of the in-house compound library for VSCs.

The results of analysis are presented in Table 4. Among 20 analyzed VSCs, only 10 were present in authentic samples of brandy of different origins, and only thiophenol was detected in every studied sample. In pear brandy, 6 different VSCs were detected, and among them, 2-thiophenecarboxaldehyde was present in the largest quantity (3.39 µg/L), followed by 1-butanethiol (1.17 µg/L). Moreover, pear brandy contained trace levels of dipropyl and dibutyl sulfide and thiophenol. In the analyzed apple brandy, only 4 VSCs were detected; however, the concentration of 2-methyltetrahydrothiophene-3-one was relatively high, and for that reason, the total concentration of VSCs found in this sample was the highest and reached almost 82 µg/L. The lowest concentration of compounds of interests was found in plum brandy, where only dimethyl disulfide was found in more than trace concentration.

Very few authors have analyzed the presence of VSCs in spirit beverages. Ledauphin et al. [10] analyzed freshly distilled Calvados and Cognac samples with use of sulfur chemiluminescence detector. They found that 3-thiophenecarboxaldehyde and 2-thiophenecarboxaldehyde are the compounds present in highest quantities, whereas Cognac contains 5–6 folds more of them than Calvados. The authors also found significant concentrations of ethyl propyl sulfide, ethyl propyl disulfide, and dibutyl sulfide. Campillo et al. [4] studied the presence of dimethyl sulfide, methyl propyl sulfide, and dimethyl disulfide in different fermented beverages, including whisky. Four different samples of whisky were tested and in three of them, authors found from 0.66 to 1.18 ng/mL of dimethyl disulfide; in the fourth sample methyl propyl sulfide was determined (0.28 ng/mL).

## 3. Materials and Methods

### 3.1. Chemicals

The studied sulfur compounds: ethanethiol (97%), dimethyl sulfide (99%), 1-propanethiol (99%), thiophene (99%), diethyl sulfide (98%), 1-butanethiol (99%), dimethyl disulfide (99%), ethyl thioacetate (98%), 1-pentanethiol (98%), 2-(methylthio)ethanol (99%), 3-(methylthio)propionaldehyde (96%), dipropyl sulfide (97%), diethyl disulfide (99%), thiophenol (97%), 2-methyltetrahydrothiophene-3-one (97%), 3-thiophenecarboxaldehyde (98%), 3-(methylthio)-1-propanol (98%), 2-thiophenecarboxaldehyde (98%), ethyl 3-(methylthio)propionate (99%), dibutyl sulfide (98%), dipropyl disulfide (98%), and benzothiazole (96%) were purchased from Sigma-Aldrich (St. Louis, MO, USA). Ethanol and sodium chloride were obtained from Chempur (Piekary Slaskie, Poland). EDTA was purchased from Sigma-Aldrich (St. Louis, MO, USA). Distilled water was purified with use of Millipore Simplicity UV purification system (18.2 MΩ·cm, Simplicity Millipore Waters, Milford, MA, USA).

### 3.2. SPME Equipment and HS–SPME Optimization

The stock mixture of standard solutions was prepared by addition of 20 µL of each of the VSCs to 96% *v*/*v* ethanol in the total volume of 25 mL. The working mixture was prepared by 10-fold dilution of stock mixture in 96% *v*/*v* ethanol. The internal standard (IS) solution (thiophene) was prepared in the concentration of 9.42 mg/L. Samples for the method development were prepared in 10 mL of total ethanol–water solution volume, and 10 µL of working mixture along with 10 µL of IS was added to each tested sample.

To find the best SPME conditions, a number of variables were tested, i.e., fiber coating, ethanol concentration, addition of NaCl, addition of EDTA, and temperature and time of extraction.

Five different fiber coatings from Supelco (Bellefonte, PA, USA) were tested: 100 μm Polydimethylsiloxane (PDMS), 60 µm Carbowax/Polyethylene Glycol (PEG), 85 μm polyacrylate (PA), 85 μm Carboxen/PDMS (CAR/PDMS), and 50/30 μm DVB/Carboxen/PDMS (DVB/CAR/PDMS). All tested fibers were of 1 cm length. Before injection, fibers were conditioned in the hot injection port according to manufacturer’s recommendations. Additionally, fibers were also conditioned, before each sample extraction, for 10 min at 200 °C (PEG) or 250 °C (PA, PDMS, CAR/PDMS, DVB/CAR/PDMS).

To examine the effect of ethanol concentration on the extraction of VSCs, the mixtures of ethanol and water were prepared in the total volume of 10 mL with final ethanol concentration of 2.5%, 5%, and 10% *v*/*v*. The effect of NaCl addition was tested in the range of 0–3 g of NaCl per sample, which corresponds to a concentration of 0–30% *w*/*v*. The EDTA was added to selected samples in a dosage of 0.1 g. The time and temperature of extraction were tested in values of 15, 30, and 45 min, and 20, 35, 50, 65, and 75 °C, respectively.

Each sample was prepared in a 20 mL glass screw-cap vial with magnetic cap and teflon/silicone septa and equilibrated for 15 min at the desired temperature. The agitation rate of 600 rpm was constant for all analyses. After extraction, the analytes were desorbed in the GC injection port from the fiber at 250 °C for 5 min. Samples were analyzed in triplicate.

### 3.3. Sample Preparation

Three samples of commercial brandies were analyzed: pear brandy (alcohol content 40% *v*/*v*), plum brandy (alcohol content 70% *v/v*), and apple brandy (alcohol content 40% *v*/*v*). Before extraction, samples were diluted with high-purity deionized water to alcohol content of 2.5% *v*/*v* in the total volume of 10 mL in 20 mL screw-cap glass vials, and 2 g of NaCl (20% *w*/*v*) along with 0.1 g of EDTA (1% *w*/*v*) were added to each sample.

### 3.4. Gas Chromatography–Mass Spectrometry (GC–MS) Analysis

Analyses were carried out using Agilent 7890A (Agilent Technologies, Santa Clara, CA, USA) coupled to a mass spectrometer (Agilent MSD 5975C, Agilent Technologies, Santa Clara, CA, USA). The separation of compounds was performed on DB-1ms capillary column (30m × 0.25 mm × 0.25 µm, Agilent J&W, Agilent Technologies Santa Clara, CA, USA). The Topaz 0.75 mm ID SPME inlet liner (Restec) for Agilent GCs was used. Injections were performed in the splitless mode. As a carrier gas, helium was used with a flow rate of 1.1 mL/min. The oven temperature was as follows: 30 °C for 6 min, raising to 130 °C at a rate of 10 °C/min, then to 220 °C at a rate of 20 °C/min, where it was held for 2 min. The temperatures of the MS ion source, transfer line, and quadrupole analyzer were 230, 250, and 150 °C, respectively. The electron impact energy was 70 eV. The mass spectrometer was operating in the full scan mode. Qualification of VSCs in the real samples was performed by comparison of obtained spectra with the reference mass spectra from NIST/EPA/NIH mass spectra library (2012; Version 2.0 g.), with the retention times of authentic standards and confirmed with use of the deconvolution procedure. Deconvolution was done using MassHunter Workstation Software (Agilent). Quantification of VSCs in the real samples was performed using selected ion monitoring (SIM) mode. The ions monitored in SIM mode (Table S1) were selected based on data gathered in NIST/EPA/NIH Mass spectra library (2012; Version 2.0 g.). For high selectivity, ions of VSC were grouped and monitored in the time segments. The total dwell time ranged between 60 and 120 ms, with an acquisition rate higher than 6 cycles/sec. Agilent MassHunter software (Agilent Technologies, Santa Clara, CA, USA) was used for data processing.

## Figures and Tables

**Table 1:** The effect of ethanol (% *v*/*v*) and NaCl (% *w*/*v*) concentration on the response of individual tested compounds. Data expressed as percent of maximum value obtained for each compound.

Compound	Ethanol	2.5%				5%				10%			
	NaCl	0	10	20	30	0	10	20	30	0	10	20	30
Ethanethiol		12.3	14.5	100.0	0.0	0.0	0.0	0.0	0.0	0.0	0.0	0.0	0.0
Dimethyl-sulfide		25.7	28.7	100.0	0.0	0.0	0.0	0.0	0.0	0.0	0.0	0.0	0.0
1-Propanethiol		65.2	43.1	100.0	59.5	17.7	22.6	13.9	10.9	10.4	5.7	6.3	3.8
Thiophene		71.8	55.6	100.0	64.7	18.0	42.8	35.4	28.8	13.4	22.0	27.7	14.3
Diethyl-sulfide		58.9	48.0	100.0	76.0	14.2	38.2	50.5	26.5	19.1	19.4	16.6	11.9
1-Butanethiol		80.2	53.5	100.0	74.8	22.0	32.2	48.5	25.4	30.6	19.4	22.3	11.7
Dimethyl-disulfide		56.0	50.1	100.0	81.5	15.4	37.0	69.8	46.7	18.7	52.5	18.5	16.4
Ethyl-thioacetate		49.9	50.8	100.0	91.3	15.0	32.7	51.6	53.9	15.2	28.6	30.7	38.4
1-Pentanethiol		85.2	68.3	100.0	71.1	39.0	34.5	48.9	40.9	38.9	38.4	43.9	25.3
Dipropyl-sulfide		89.8	78.3	100.0	80.5	43.5	53.6	61.0	51.0	38.3	49.5	50.8	33.4
Diethyl-disulfide		80.5	74.8	100.0	80.3	39.5	53.5	64.8	54.4	33.4	48.3	49.3	35.8
Thiophenol		100.0	2.1	2.2	2.8	52.0	1.0	1.2	1.6	31.5	1.1	0.5	0.9
2-Methyltetrahydrothiophene-3-one		86.9	50.9	88.6	100.0	30.3	28.9	47.7	50.6	16.9	27.0	39.1	39.3
3-Thiophenecarboxaldehyde		32.1	48.1	100.0	96.6	12.6	33.1	74.3	82.1	9.9	28.6	38.2	51.1
2-Thiophenecarboxaldehyde		47.1	55.0	90.8	100.0	23.6	40.1	69.8	89.0	19.6	32.9	37.7	55.5
Ethyl-3-(methylthio)propionate		24.6	40.7	80.2	100.0	13.1	29.0	59.6	69.9	9.5	27.8	33.4	50.5
Dibutyl-sulfide		100.0	83.5	96.4	82.7	65.2	70.1	75.2	69.1	68.3	70.9	77.8	49.4
Dipropyl-disulfide		100.0	86.4	99.3	85.2	65.2	72.3	79.0	70.9	66.9	73.0	79.0	50.2
Benzothiazole		36.7	51.5	86.9	100.0	18.5	53.5	64.5	87.2	7.2	19.3	29.1	34.3

**Table 2:** The effect of extraction temperature on the response of individual tested compounds. Data expressed as percent of maximum value obtained for each compound.

Compound	Temperature (°C)				
	20	35	50	65	75
Ethanethiol	100.0	32.2	14.4	0.0	0.0
Dimethyl-sulfide	100.0	42.0	29.9	0.0	0.0
1-Propanethiol	100.0	41.3	37.7	5.8	2.6
Thiophene	100.0	83.7	59.5	11.6	6.6
Diethyl-sulfide	100.0	75.3	47.9	10.4	6.9
1-Butanethiol	100.0	79.3	47.7	14.6	6.3
Dimethyl-disulfide	100.0	88.6	57.4	18.5	5.7
Ethyl-thioacetate	100.0	89.9	69.5	31.1	16.1
1-Pentanethiol	100.0	88.2	58.6	18.8	8.7
Dipropyl-sulfide	100.0	99.0	77.5	30.8	14.6
Diethyl-disulfide	97.2	100.0	80.8	36.9	18.3
Thiophenol	80.3	100.0	85.3	48.9	34.3
2-Methyltetrahydrothiophene-3-one	63.2	100.0	89.7	58.9	31.7
3-Thiophenecarboxaldehyde	30.7	64.1	100.0	69.6	54.7
2-Thiophenecarboxaldehyde	28.2	64.3	100.0	78.9	59.9
Ethyl-3-(methylthio)propionate	20.2	57.5	100.0	54.0	42.5
Dibutyl-sulfide	87.9	97.0	100.0	66.3	41.6
Dipropyl-disulfide	86.3	97.2	100.0	69.8	44.1
Benzothiazole	20.6	37.5	69.6	100.0	86.9

**Table 3:** Limits of detection and quantification, linearity, and recovery of the method.

Compound	R^2^	LOQ (µg/L)	LOD (µg/L)	Range of assayed concentration (µg/L)		Linearity (µg/L)		Recovery (%)	
				min	max	min	max	min	max
Ethanethiol	0.9950	0.569	0.171	0.04	40.77	0.64	10.19	89.91	113.03
Dimethyl-sulfide	0.9904	0.208	0.063	0.04	37.84	0.59	9.46	78.02	107.77
1-Propanethiol	0.9894	0.611	0.183	0.06	60.97	0.95	15.25	75.71	176.99
Diethyl-sulfide	0.9972	0.081	0.024	0.07	69.23	0.08	34.62	96.66	109.41
1-Butanethiol	0.9319	0.011	0.003	0.07	67.27	0.53	16.82	87.24	133.87
Dimethyl-disulfide	0.9947	0.009	0.003	0.09	94.60	0.09	94.70	96.48	109.44
Ethyl thioacetate	0.9924	0.002	0.001	0.08	86.65	0.08	43.32	93.99	106.68
1-Pentanethiol	0.9640	0.014	0.004	0.07	70.93	0.14	8.87	86.11	129.13
Dipropyl-sulfide	0.9954	0.146	0.044	0.07	72.95	0.15	36.48	97.68	105.53
Diethyl-disulfide	0.9932	0.116	0.035	0.09	89.56	0.12	11.19	89.01	105.88
Thiophenol	0.9969	0.048	0.015	0.09	94.41	0.09	23.60	102.08	122.60
2-Methyltetrahydrothiophene-3-one	0.9991	0.045	0.014	0.11	108.57	0.11	108.57	92.48	108.62
3-Thiophenecarboxaldehyde	0.9984	0.053	0.016	0.11	114.68	0.11	57.34	90.37	110.20
2-Thiophenecarboxaldehyde	0.9983	0.208	0.063	0.11	111.71	0.21	55.85	92.89	106.75
Ethyl 3-(methylthio)propionate	0.9943	0.187	0.056	0.09	95.86	0.09	47.93	99.94	109.93
Dibutyl-sulfide	0.9957	0.103	0.031	0.07	75.60	0.10	37.80	92.89	112.25
Dipropyl-disulfide	0.9957	0.132	0.040	0.08	81.55	0.13	10.19	94.36	110.31
Benzothiazole	0.9945	0.208	0.062	0.10	107.10	0.21	53.55	112.95	125.63

**Table 4:** Average concentration (µg/L) of VSCs found in tested brandy samples.

Compound	Molecular Formula	Retention Time	Pear Brandy	Plum Brandy	Apple Brandy
Ethanethiol	C_2_H_6_S	1.56	<LOD	<LOD	<LOD
Dimethyl-sulfide	C_2_H_6_S	1.626	<LOD	<LOD	<LOD
1-Propanethiol	C_3_H_8_S	2.177	<LOD	<LOD	<LOD
Diethyl-sulfide	C_4_H_10_S	3.555	<LOD	<LOD	<LOD
1-Butanethiol	C_4_H_10_S	3.815	1.17±0.06	<LOD	<LOD
Dimethyl-disulfide	C_2_H_6_S_2_	4.521	<LOD	0.14 ± 0.03	0.11 ± 0.03
Ethyl thioacetate	C_4_H_8_OS	5.57	<LOD	<LOD	<LOD
1-Pentanethiol	C_5_H_12_S	7.568	<LOD	<LOD	<LOD
Dipropyl sulfide	C_6_H_14_S	9.895	<LOQ	<LOD	<LOD
Diethyl disulfide	C_4_H_10_S_2_	10.478	<LOD	<LOQ	<LOD
Thiophenol	C_6_H_6_S	11.292	<LOQ	<LOQ	<LOQ
2-Methyltetrahydrothiophene-3-one	C_5_H_8_OS	11.539	<LOD	<LOD	81.76 ± 1.06
3-Thiophenecarboxaldehyde	C_5_H_4_OS	11.578	<LOD	<LOD	<LOD
2-Thiophenecarboxaldehyde	C_5_H_4_OS	11.785	3.39 ± 0.17	<LOD	<LOD
Ethyl 3-(methylthio)propionate	C_6_H_12_O_2_S	13.941	<LOD	<LOD	<LOQ
Dibutyl sulfide	C_8_H_18_S	14.007	<LOQ	<LOD	<LOD
Dipropyl disulfide	C_6_H_14_S_2_	14.254	<LOQ	<LOQ	<LOD
Benzothiazole	C_7_H_5_NS	15.839	<LOD	<LOD	<LOD

## Supplementary Materials

The following are available online: Table S1: SIM ions used in the GC–MS analysis.
