# Supplementary material for: Development of the Method for Determination of Volatile Sulfur Compounds (VSCs) in Fruit Brandy with the Use of HS–SPME/GC–MS
Source: Molecules. 2020 Mar 9;25(5):1232. doi: 10.3390/molecules25051232 (PMC7179427; doi:10.3390/molecules25051232)
Supplement: Supplementary file 1 [file molecules-25-01232-s001.pdf]

molecules

Supplementary materials

Table S1: SIM ions used in the GC-MS analysis

Compound	Quantitation ion *m/z*	Confirmation ions *m/z*
**Ethanethiol**	62	61
**Dimethyl sulfide**	62	61
**1-Propanethiol**	76	43, 47
**Diethyl sulfide**	75	47, 90
**1-Butanethiol**	41	56, 90
**Dimethyl disulfide**	94	45, 79
**Ethyl thioacetate**	43	45, 104
**1-Pentanethiol**	42	55, 70
**Dipropyl sulfide**	43	89, 118
**Diethyl disulfide**	122	66, 29
**Thiophenol**	110	66, 109
**2-Methyltetrahydrothiophene-3-one**	60	45, 116
**3-Thiophenecarboxaldehyde**	111	39, 112
**2-Thiophenecarboxaldehyde**	111	39, 112
**Ethyl 3-(methylthio)propionate**	74	61, 148
**Dibutyl sulfide**	61	29, 56
**Dipropyl disulfide**	43	108, 150
**Benzothiazole**	135	69, 108
